# Extramedullary Hematopoiesis: An Unusual Finding in Subdural Hematomas

**DOI:** 10.1155/2011/718585

**Published:** 2011-09-27

**Authors:** Rong Li, Vishnu V. B. Reddy, Cheryl Ann Palmer

**Affiliations:** ^1^Department of Pathology, University of Alabama at Birmingham, Birmingham, AL 35294, USA; ^2^Department of Neurology, University of Alabama at Birmingham, 1960 6th Avenue South, PD6A 175E, Birmingham, AL 35294, USA

## Abstract

We present a case of a 59-year-old man who was found to have clusters of hyperchromatic, small, round nucleated cells within a subdural hematoma removed after a skull fracture. Immunohistochemistry study confirmed that the cells were hematopoietic components predominantly composed of normoblasts. In this paper, we describe the clinical and pathological findings. A brief review of published information on extramedullary hematopoiesis in subdural hematoma and the mechanisms of pathogenesis are also discussed. While extramedullary hematopoiesis is seen anecdotally by neuropathologists in chronic subdural hematomas, only a few cases are documented in the literature. Furthermore, extramedullary hematopoiesis in subdural hematoma can pose a diagnostic challenge for general pathologists who encounter subdural hematoma evacuations seldom in their surgical pathology practices.

## 1. Introduction

Subdural hematomas are a common posttraumatic finding and are caused by tearing of cortical bridging veins. They can also be seen in patients with underlying coagulopathies or other hematopoietic disorders. These hematomas can resolve spontaneously if small. Surgical evacuation is required to relieve pressure when they are large. On most occasions, the evacuated specimen consists of clotted blood, macrophages (if subacute), with collagen, neovascularization, and focal hemosiderin (if chronic). We present a case of a subdural hematoma with foci of hematopoietic components largely made up of normoblasts. Hematopoietic elements identified outside of the bone marrow are defined as extramedullary hematopoiesis (EMH). EMH is normally seen during embryonic development and fetal life. However, occurrence of EMH after this period of life is considered abnormal.

## 2. Case Report

This 59-year-old man had a past medical history of diabetes, hypertension, and pancreatitis. He sustained a fall from a 12-foot ladder in December, 2009. At that time, he was diagnosed with subarachnoid hemorrhage in the right interhemispheric fissure, a right minimal frontal bone fracture involving the frontal sinus, a right frontal contusion and a right open femur fracture. No operational interventions were given to the patient for his skull and facial fractures since the fractures were minimally displaced. His right femur fracture was treated with intramedullary nail fixation. His hospital course was uneventful. About a month later, he was discharged to a local rehabilitation center and was continuously treated with anticoagulants for femoral deep vein thrombosis. Shortly thereafter, he presented to the emergency department with altered metal status after a fall from bed. Computed tomography of the brain showed a right acute upon chronic subdural hematoma. A left frontotemporal craniotomy with evacuation of a subdural hematoma was performed. The patient was discharged to home without complications.

**Figure 1:**
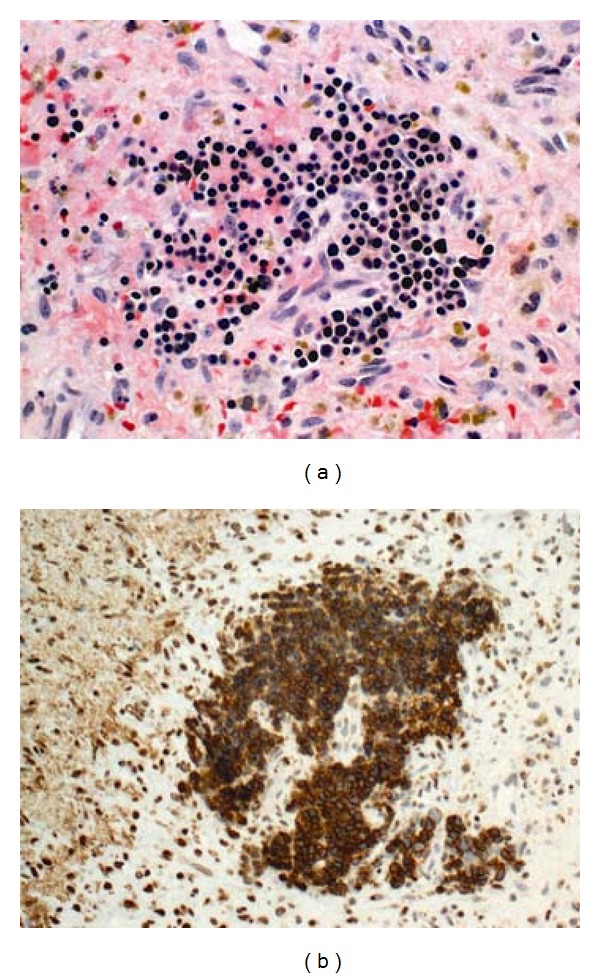
EMH within subdural hematoma. (a) Clusters of small round cells with hyperchromatic round nuclei and scant cytoplasm in a background of connective tissue with acute and chronic hemorrhage (H and E ×400). (b) The small round cells show strong immunoreactivity for glycophorin A, a marker for erythroblastic line cells (glycophorin A ×400).

Microscopic examination of the hematoma demonstrated neovascularized tissue containing both acute and chronic inflammation and hemosiderin deposition consistent with a subdural membrane. Remarkably, there were numerous microscopic nodules of hyperchromatic, small, round cells scattered diffusely in the hematoma (Figure 1(a)), which were positive for glycophorin (Figure 1(b)), an erythrocyte precursor marker. The cells of interest were negative for the B-cell marker CD20, endothelial cell marker CD34 and were mostly negative for the T-cell marker CD3. These findings are diagnostic of EMH with primarily erythroid precursors in a subdural hematoma.

## 3. Discussion

It is generally believed that EMH is a compensatory process associated with anemia and other bone marrow diseases which leads to chronic hematopoietic deficiency. EMH sites in adults are most commonly liver, spleen, and lymph nodes. However, EMH can occur in any site including the central nervous system. Intracranial EMH is a rare event and is most frequently associated with thalassemia and myelofibrosis [1]. The cranial dura, especially the falx, is the most commonly involved site. The first report of EMH in a subdural hematoma was published in 1966 [2] in a 4-month old infant who was very anemic and presented with an enlarged head with a persistent subdural hematoma. Nucleated red blood cells were identified within the hematoma, but not in the peripheral blood, and they disappeared shortly thereafter even with the continuous presence of the subdural hematoma. The authors proposed that the occurrence of subdural EMH in this case was either a congenital anomaly or a secondary reaction to the child's anemia. There have been only a few subsequent reports of patients presenting with subdural hematoma-associated EMH [3–5]. None of these reports included patients with a clinical history of skull fracture as was seen in our case. The clinical significance of the EMH in subdural hematoma is thought to be of no importance as concluded in one previous report [3]. 

There are a few theories of the mechanisms of EMH [6]. When the bone marrow space is inadequate as a result of myelofibrosis or bone metastasis, EMH occurs as a compensatory phenomenon with increased numbers of circulating hematopoietic stem cells. Another theory is the myelostimulatory theory, which proposes that EMH presents in fetal hematopoietic sites under stimulation of unknown myelostimulatory factors. Koch et al. [6] also hypothesized another possible explanation of EMH, the “redirected differentiation theory.” They proposed that stem cells of different tissue types may differentiate into hematopoietic stem cells when induced by unknown circulating factors in response to anemia or other hematologic disorders. However, these above theories fail to explain all cases of EMH. Very rarely, EMH occurs following a traumatic event as seen in our case. This phenomenon has been observed in lung tissue following bone fracture or cardiac surgery and in the presacral area following a sacrum fracture [7]. It is presumed that the origin of EMH in these cases is from bone marrow embolism. In the present case, the patient had no underlying hematopoietic disorder and developed a subdural hematoma following head trauma and a skull fracture. It is highly possible that a small nidus of marrow hematopoietic cells migrated from the fracture site and leaked into the subdural space with resultant EMH within the hematoma. Interestingly, subdural hemorrhage/hematoma can be a secondary process of EMH in patients with hematopoietic disorders [8, 9].

On most occasions, EMH in subdural hematoma is an incidental finding. However, the clusters of nucleated red blood cells can present a diagnostic pitfall for surgical pathologists as the common differential diagnoses of groups of small round cells with hyperchromatic nuclei and scant cytoplasm in the subdural space include metastatic malignant tumors, such as small cell carcinoma, melanoma, and/or lymphoma. However, negative immunostains for epithelial, neuroendocrine, mesenchymal, and lymphoid markers help to rule out these possibilities. The final diagnosis of EMH can easily be confirmed by staining the small round cell clusters which are immunoreactive for the erythrocyte marker glycophorin.

## 4. Conclusion

In summary, we present a rare case of EMH in a subdural hematoma in a patient with head trauma and a skull fracture, but no evidence of an underlying myeloproliferative disorder. This case supports the hypothesis of bone marrow embolism as the origin of EMH. In addition, we report this case to help to minimize the chance of misdiagnosis when surgical pathologists encounter EMH at an unanticipated site or under unexpected circumstances.
